# Depression and anxiety in relation to catechol-O-methyltransferase Val^158^Met genotype in the general population: The Nord-Trøndelag Health Study (HUNT)

**DOI:** 10.1186/1471-244X-8-48

**Published:** 2008-06-25

**Authors:** Petter M Bækken, Frank Skorpen, Eystein Stordal, John-Anker Zwart, Knut Hagen

**Affiliations:** 1 Department of Neuroscience, Faculty of Medicine, Norwegian University of Science and Technology, Trondheim, Norway; 2 Department of Laboratory Medicine, Children's and Women's Health, Faculty of medicine, Norwegian University of Science and Technology, Trondheim, Norway; 3 Norwegian National Headache Centre, Section of Neurology, St. Olavs' Hospital, Trondheim, Norway; 4 National Centre for Spinal Disorders, St. Olavs' Hospital, Trondheim, Norway; 5 Department of Neurology, Ullevål University Hospital, Oslo, Norway; 6 Department of psychiatry, Hospital Namsos, Namsos, Norway

## Abstract

**Background:**

The catechol-O-methyltransferase (COMT) gene contains a functional polymorphism, Val158Met, which has been linked to anxiety and depression, but previous results are not conclusive. The aim of the present study was to examine the relationship between the Val158Met COMT gene polymorphism and anxiety and depression measured by the Hospital Anxiety and Depression Scale (HADS) in the general adult population.

**Methods:**

In the Nord-Trøndelag Health Study (HUNT) the association between the Val158Met polymorphism and anxiety and depression was evaluated in a random sample of 5531 individuals. Two different cut off scores (≥ 8 and ≥ 11) were used to identify cases with anxiety (HADS-A) and depression (HADS-D), whereas controls had HADS-A <8 and HADS-D <8.

**Results:**

The COMT genotype distribution was similar between controls and individuals in the groups with anxiety and depression using cut-off scores of ≥ 8. When utilizing the alternative cut-off score HADS-D ≥ 11, Met/Met genotype and Met allele were less common among men with depression compared to the controls (genotype: p = 0.017, allele: p = 0.006). In the multivariate analysis, adjusting for age and heart disease, depression (HADS-D ≥ 11) was less likely among men with the Met/Met genotype than among men with the Val/Val genotype (OR = 0.37, 95% CI = 0.18–0.76).

**Conclusion:**

In this population-based study, no clear association between the Val158Met polymorphism and depression and anxiety was revealed. The Met/Met genotype was less likely among men with depression defined as HADS-D ≥ 11, but this may be an incidental finding.

## Background

The catechol-O-methyltransferase (COMT) gene located on chromosome 22q11.2 contains a common functional polymorphism at codon 158. This polymorphism has been subject to a large number of studies, due to the COMT enzyme's important role in the metabolism of catecholamines (1). A substitution of valine (Val) by methionine (Met) at codon 158 affects the activity of the COMT enzyme, and individuals with the Val/Val genotype have a 3–4-times higher enzyme activity than those with the Met/Met genotype [1]. Since the alleles are co-dominant, heterozygous individuals (Val/Met) show an enzyme activity halfway between the homozygous genotypes.

The Val158Met polymorphism has been linked to a broad range of psychiatric disorders including anxiety and mood disorders [2]. Although anxiety and depression both are influenced by several genes in combination with environmental factors, association studies with large samples of subjects are appropriate for searching susceptibility genes with minor effects on these disorders [3]. A large European multicenter study including 1512 participants reported an association between the Val/Val genotype and major depression with early onset [3], but conflicting results have been found in other studies with fewer participants [4]. Similarly, inconsistent findings have also been reported in studies evaluating the relationship between the Val158Met polymorphism and anxiety [5-7]. Ethnic heterogeneity and gender specificity may have influenced on the inconclusive results [8].

Most of the previous studies evaluating the relationship between the Val158Met polymorphism and anxiety and/or mood disorders have been case-control studies. In such studies undiagnosed- and untreated cases with depression and/or anxiety may have been missed. Generalization of the results should therefore be done with caution. Because population-based studies have the potential to intercept undiagnosed- and untreated cases [9], the influence of the Val158Met polymorphism on anxiety and depression should also be evaluated in the general population.

To the best of our knowledge, this is the first population-based study evaluating the relationship between the Val158Met polymorphism and anxiety and depression measured by the Hospital Anxiety and Depression Scale (HADS). The dimensional structure of HADS has been found stable across age-groups [9], and the HADS questionnaire is widely used to measure anxiety and depression in somatic and psychiatric patients as well as in the general population [10]. Previous studies on HADS have shown good reliability and validity compared to other symptom scales of depression and anxiety [10-15].

In the present study, we examined the relationship between the Val158Met polymorphism and anxiety and depression measured by HADS in a population-based cohort of 5531 subjects.

## Methods

### Study population

Between August 1995 and June 1997, all inhabitants aged 20 years or older in Nord-Trøndelag County were invited to participate in the Nord-Trøndelag Health Study (HUNT). In brief, two questionnaires including more than 200 health related questions were administrated to the participants. The population in Nord-Trøndelag County was ethnically homogenous (less than 3% of subjects were of non-Caucasian ethnicity), making it suitable for epidemiological genetic research [16].

Out of 92,936 invited individuals, a total of 65,291 subjects (70%) answered the first questionnaire and participated in a health examination. The first questionnaire included questions about e.g. vascular diseases, smoking, and physical activity [16]. The health examination included measurements of height, weight, and blood pressure, and blood samples measuring e.g. cholesterol were collected [16].

Details of the non-participants are described elsewhere [17]. Blood samples were collected from 62,664 of the study population whenever they attended, and stored at HUNT 2 Biobank [16]. In 2002, all surviving participants (n = 61,426) were asked for permission to use the blood samples in genetic analyses. Over 98% (n = 60,241) accepted this [16]. Analyses on the Val158Met polymorphism have been carried out on 5,531 participants, of whom 4513 (82%) were selected completely at random. The first 1018 (18%) subjects were randomly selected among an older group who did not have self-reported diabetes mellitus [18]. This group was generated in connection with a planned genetic study on diabetes that needed age-matched controls to a diabetic population. As a consequence, the prevalence of self-reported diabetes mellitus was somewhat lower among individuals with known genotype than among those without COMT data available (1.3% versus 3.2%, p < 0.001).

### Genotyping

DNA for genotyping was extracted from peripheral blood leukocytes from EDTA whole blood or blood clots, stored in the HUNT 2 Biobank, manually with the use of Puregene kit (Gentra Systems Inc., Minneapolis, MN) or with the Autopure LS (Gentra Systems Inc., Minneapolis, MN). The laboratory technicians were blinded for other HUNT data. Genotypes of the COMT Val158Met polymorphism were determined using a LightCycler Real Time PCR machine (Roche Diagnostics Scandinavia AB, Bromma, Sweeden) [19]. PCR-technique was carried out in 20 μL reagent on a LightCycler System by using 2 μL genomic DNA and LightCycler-FastStart DNA Master Hybridization Probes kit (Roche Diagnostics, Bromma, Sweeden). Details on PCR primers and hybridization probes used have been published elsewhere [17]. Based on melting-curve profiles, the genotypes of the participants were classified as Val/Val, Val/Met or Met/Met.

### Anxiety and depression

HADS is a self-assessment scale containing 14 questions developed to detect states of anxiety and depression, specifically designed for non-psychiatric hospital departments [20]. To each question there are four possible response options which are scored from 0 to 3 points depending on the severity of the symptoms. There are seven questions related to depressive symptoms (HADS-D subscale) and seven questions concerning anxiety symptoms (HADS-A subscale). Based on empirical knowledge a clinically significant anxiety disorder is defined by a HADS-A score of ≥ 8, while depressive disorder is defined by HADS-D ≥ 8. These limits are found to give the optimal balance between sensitivity and specificity [11]. Based on HADS-A and HADS-D scores, the participants were classified into one of five groups: 1) No disorder (controls): both HADS-A <8 and HADS-D <8; 2) Anxiety disorder: HADS-A ≥ 8; 3) Depressive disorder: HADS-D ≥ 8; 4) Combined disorder: both HADS-A ≥ 8 and HADS-D ≥ 8; and 5) Unclassified. This last group consisted of subjects with insufficient HADS score data. Some had total HADS scores but unavailable subscale scores, while others only had valid HADS-A or HADS-D score. To increase the specificity [9] and with intention identify individuals with moderate or severe disorder, analyses using cut-off score ≥ 11 were also performed, recommended for the first time 25 years ago [21]. Analyses were performed for men and women separately because previous studies have reported a gender difference regarding anxiety and the Val158Met polymorphism [8].

### Ethics

The study was approved by the Regional Committee of Ethics, by the Norwegian Data Inspectorate and by the Directorate for Health and Social Affairs.

### Statistical analyses

Baseline data were compared between genotype status with analyses of variance (one-way ANOVA) for continuous variables and with Pearson's chi-square test for categorical variables. Blood pressure values were compared with the non-parametric Mann-Whitney U test because of a skewed distribution of the data [18].

Gender comparisons for each genotype regarding HADS-A scores and HADS-D scores as continuous variables were performed with Mann-Whitney U because of skewed data. Spearman's correlation analyses were conducted to correlate scores of HADS-A and HADS-D with genotypes.

Chi-square test was used to evaluate dichotomous variables of HADS-A and HADS-D. Binary multivariate logistic regression was used to estimate odds ratios (OR) for the association between genotype and each of the two HADS subscales, i.e. depression and anxiety disorder (dependent variable). Potential confounding was evaluated by adjusting for sex, age (5-year categories) and ischemic heart disease. The precision for the OR was estimated with 95% confidence interval (CI).

Two-tailed estimations of significance were used, and the level of significance was set at p < 0.05.

Overall, our sample of 5,531 had more than 90% power to detect a 3% difference in prevalence of anxiety and depression between genotypes with 95 percent certainty.

Statistical analyses were performed using the Statistical Package for the Social Sciences (SPSS), version 14.0 (SPSS Inc, Chicago, IL).

## Results

The genotype distribution among the 5,531 individuals was in Hardy-Weinberg equilibrium. Demographic data of the different genotype groups are shown in Table 1. No significant difference in gender, age, education level, body mass index, cholesterol level, smoking status, level of physical activity, diabetes-, stroke- or headache prevalence was found between the genotype groups. However, individuals with Val/Val genotype had lower prevalence of myocardial infarction (2.3% versus 3.6%, p < 0.05) and ischemic heart disease (myocardial infarction and/or angina pectoris) (5.7% versus 7.7%, p = 0.02) compared to those with the other genotypes (Table 1). The individuals with known genotype were significantly older and had, consequently, higher blood pressure and cholesterol level, higher prevalence of ischemic heart disease and chronic musculoskeletal pain, and had lower physical activity level than those with unknown COMT genotype (Table 1).

**Table 1:** *COMT *genotypes related to sex, mean age, education > 12 years, mean systolic and diastolic blood pressure, and several factors associated with hypertension.

	No COMT genotyping	Met/Met	Val/Met	Val/Val
Characteristics	(n = 60,072)	(n = 1769)	(n = 2726)	(n = 1036)
Sex, female (%)	53.1	53.7	54.3	56.1
Age, mean (SD)	49.2 (17.3)^1^	50.7 (17.9)	50.7 (18.0)	50.2 (17.7)
Education > 12 years (%)	19	18	18	18
Median systolic blood pressure (mmHg)	134^1^	136.5	136	136
Median diastolic blood pressure (mmHg)	79^1^	80	80	80
Body mass index, kg/m^2 ^(SD)	26.4 (4.1)	26.4 (4.1)	26.3 (4.1)	26.3 (4.2)
Cholesterol, mmol (SD)	5.89 (1.26)^1^	5.96 (1.30)	5.96 (1.30)	6.02 (1.33)
Current smoking (%)	28.2	27.9	28.4	29.6
High level of physical activity (%)	15.8^1^	13.9	14.1	14.8
Diabetes mellitus (%)	3.2^1^	1.3	1.3	1.1
Myocardial infarction (%)	3.3	3.8	3.4	2.3^2^
Angina pectoris (%)	5.0^1^	6.7	5.8	5.1
Stroke (%)	1.9	2.1	1.8	1.8
Headache (%)	38.6	39.7	40.0	37.9
Chronic musculoskeletal pain (%)	46.3^1^	46.4	50.5	48.6

1. Genotyped versus not genotyped: p < 0.003.

2. Val/Val versus other genotypes: p = 0.046.

Heart disease (myocardial infarction and/or angina pectoris):

Val/Val versus other genotypes: p = 0.02 Genotyped versus not genotyped: p < 0.02

**Figure 1:**
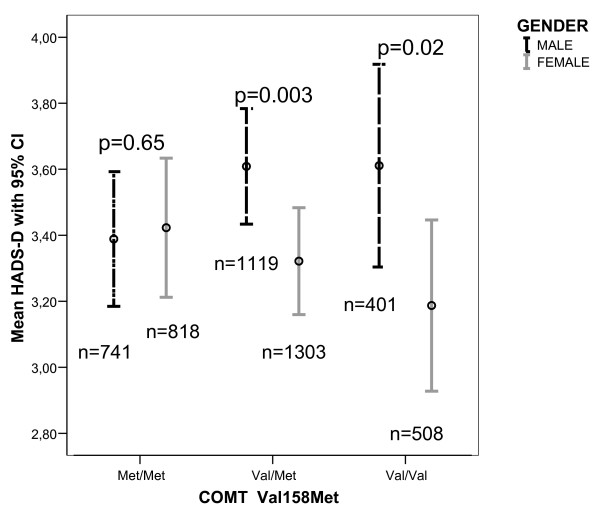
**Mean HADS-D scores with 95% confidence interval related to *COMT *genotype in men and women.** Numbers of participants are given. Gender comparison for each genotype was performed with Mann-Whitney U test.

**Figure 2:**
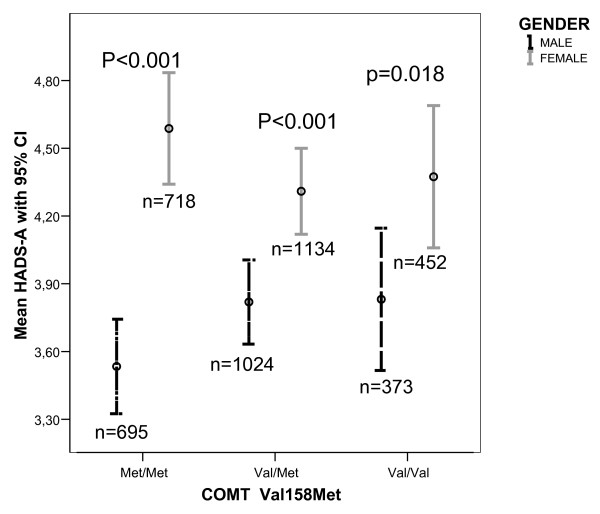
**Mean HADS-A scores with 95% confidence interval related to *COMT *genotype in men and women.** Numbers of participants are given. Gender comparison for each genotype was performed with Mann-Whitney U test.

Mean HADS-D and HADS-A scores related to genotype are displayed in Figure 1 and in Figure 2, respectively. As demonstrated, the genotype distribution of the scores tended to differ by gender, most prominent for HADS-A. However, no significant correlations between genotypes and scores of HADS-A and HADS-D were found for men or women.

The distribution of genotypes and alleles of the Val158Met polymorphism by gender failed to detect a difference between controls and subjects with anxiety and depression using the cut-off score ≥ 8 for HADS-A and HADS-D. When utilizing the alternative cut-off score ≥ 11, the Met/Met genotype and Met allele were less common among men with depression compared to the controls (genotype: 18.3% versus 33.9%, p = 0.017; allele: 46.5% versus 58.1%, p = 0.006) (Table 2). In the multivariate analysis, adjusting for age and heart diseases, HADS-D ≥ 11 was less likely among men with the Met/Met genotype than among men with the Val/Val genotype (OR = 0.37, 95% CI = 0.18–0.76). For women, this tendency was less clear (OR = 0.89, 95% CI 0.48–1.63). However, for both genders combined, depression was less common for the Met/Met genotype (OR = 0.60, 95% CI = 0.38–0.95).

**Table 2:** Distribution of HADS-score related to genotype of *COMT *in men and women.

	Met/Met		Val/Met		Val/Val	
	No.	%	No.	%	No.	%
**Genotypes**						
**Men**						
Controls (HADS-D< 8 + HADS-A < 8)	579	**33.9**	829	**48.5**	301	**17.6**
HADS-D ≥ 8	71	30.2	122	51.9	42	17.9
HADS-D ≥ 11^1^	13	18.3	40	56.3	18	25.4
HADS-A ≥ 8	70	29.7	123	52.1	43	18.2
HADS-A ≥ 11	16	25.0	39	60.9	9	14.1
Combined (HADS-D ≥ 8+HADS-A ≥ 8)	30	30.9	53	54.6	14	14.4
Unclassified (incomplete data)	29	29.5	226	51.6	83	18.9
**Women**						
Controls (HADS-D< 8 + HADS-A < 8)	549	**30.6**	889	**49.5**	358	**19.9**
HADS-D ≥ 8	96	35.2	132	48.4	45	16.5
HADS-D ≥ 11^1^	27	32.9	37	45.1	18	22.0
HADS-A ≥ 8	125	34.2	175	47.8	66	18.0
HADS-A ≥ 11	46	37.4	53	43.1	24	19.5
Combined (HADS-D ≥ 8+HADS-A ≥ 8)	47	37.3	55	43.7	24	19.0
Unclassified (incomplete data)	227	32.4	338	48.2	136	19.4
**Alleles**	**Met**		**Val**			
**Men**						
Controls	1987	**58.1**	1431	**41.9**		
HADS-D ≥ 8	264	56.2	206	43.8		
HADS-D ≥ 11^2^	66	46.5	76	53.5		
HADS-A ≥ 8	263	55.7	209	44.3		
HADS-A ≥ 11	71	55.5	57	44.5		
Combined (HADS-D ≥ 8+HADS-A ≥ 8)	113	58.2	81	41.8		
Unclassified (incomplete data)	484	55.3	392	44.7		
**Women**						
Controls	1987	**55.3**	1605	**44.7**		
HADS-D ≥ 8	324	59.3	222	40.7		
HADS-D ≥ 11^2^	91	55.5	73	44.5		
HADS-A ≥ 8	425	58.1	307	41.9		
HADS-A ≥ 11	145	58.9	101	41.1		
Combined (HADS-D ≥ 8+HADS-A ≥ 8)	49	59.1	103	40.9		
Unclassified (incomplete data)	792	56.5	610	43.5		

1. Male cases with HADS-D ≥ 11 versus male controls: p = 0.01.

2. Male cases with HADS-D ≥ 11 versus male controls: p = 0.006

## Discussion

This is the first population-based study evaluating the relationship between HADS and the Val158Met polymorphism. In a group of 5,531 unselected adults, no significant association was found with anxiety and depression using the cut-off score ≥ 8 for HADS-A and HADS-D.

When employing the alternative cut-off score ≥ 11, depression was less likely among men with the Met/Met genotype than among men with the Val/Val genotype. Val/Val individuals would be expected to have lower transsynaptic catecholamine levels, due to their high COMT enzyme activity. Such depletion of transsynaptic catecholamine neurotransmitters, like dopamine and noradrenaline, may very well influence susceptibility to depression in this genotype group [3]. Because this association was based on a very small number of cases, it may be incidental, and therefore needs to be interpreted with some caution. Interestingly, however, an association between Val/Val genotype and major depression with early onset has been reported in a multicenter study of various European populations [3]. In contrast to these findings, Ohara et al. found higher frequencies of the Met allele in depressed Asian patients compared with a control group [4]. However, because differences in COMT genotype distribution between ethnic groups are considerable, a finding in one group might not be valid for other groups [23].

Our failure to establish a significant association between anxiety and the Val158Met polymorphism is supported by previous research on various forms of anxiety [6,24], including a meta-analysis on panic disorder [8]. Conflicting results have been found in other studies. Most of these reported an association between Val/Val genotype and anxiety and panic disorder [5,25-27], whereas Woo et al. showed a Met/Met genotype predominance in patients with panic disorder [7].

The strength of this study was that our sample was drawn from a large and unselected population, which consisted of ethnically homogenous Norwegian individuals. Because no information about ethnic backgrounds was available in our data file, the relatively few numbers of non-Caucasians subjects could not be excluded in the statistical analyses. Because the distribution of the two COMT alleles differs between different ethnic groups [23], the risk of bias caused by ethnical admixture could not be ruled out.

The fact that psychiatric questions were not the primary objectives of HUNT made interest-related bias less likely. Some research has indicated that people with psychiatric disorders are over-represented among non-responders in studies of the general population [28]. This could be a weakness, since 30% of invited individuals did not attend [16]. But according to a study on non-attendants, health related reasons for not attending were unlikely among individuals below 70 years of age [17]. Our sample of more than 4000 individuals should have sufficient power to detect a difference of clinical interest in prevalence of depression and anxiety between genotypes. Because of the large sample size, we had enough power to present separate analyses by gender.

HADS, used in many former studies of the HUNT population [20,29-31], has good reliability and validity compared with other symptom scales of depression and anxiety [10-15]. Several articles have stated its suitability in samples from the general population with the potential to intercept undiagnosed- and untreated cases with depression and/or anxiety [9,11,15]. Such cases may have been missed in previous case-control studies examining the relationship between the Val158Met polymorphism and anxiety and/or mood disorders [3-8,24,25,32,33]. In addition, diagnostic differences also exist, because most previous studies have selected cases according to DSM-IV or ICD-10 categories. However, the use of rigid diagnostic criteria may fail to capture a potential genetic predisposition, and more and more studies have taken this into account by also looking at psychological traits or intermediate phenotypes instead of standard diagnoses [34-36]. HADS based diagnoses of depression and anxiety disorder do not correspond exactly to any of the specific diagnoses of DSM-IV or ICD-10, but HADS includes certain ICD-10 features [11,20]. In particular, HADS-D focuses on anhedonia, by some viewed as a core symptom of depression [14], while the HADS-A subscale mainly captures features of generalized anxiety [21]. Recently, it has been suggested that HADS-A covers two dimensions, i.e. negative affectivity and autonomic arousal [37]. A separate evaluation of these two dimensions was not possible, and as a consequence, we can not rule out a possible positive association between the Val158Met polymorphism and negative affectivity or autonomic arousal, respectively.

Our study could not confirm any clear association between the Val158Met polymorphism and anxiety or depression measured by HADS. However, a number of functional polymorphisms in- or close to the COMT gene have been described, and some recent studies have looked at multiple single nucleotide polymorphisms (SNPs) and different COMT haplotypes in relation to psychiatric phenotypes [38,39], and interaction between the COMT gene and other genes [26,33]. Due to lack of financial- and personnel resources we did not assess additional SNPs within the COMT gene or in other genes. As a consequence, we were unable to evaluate the influence of haplotypes of the COMT gene or possible interactions with other genes on HADS.

## Conclusion

In this population-based study on 5531 unselected Norwegian adults no clear association between the Val158Met polymorphism and depression and anxiety was revealed. Among men the Met/Met genotype was associated with lower prevalence of depression when using a high cut-off score, but this may be an incidental finding.

## Abbreviations

COMT: Catechol-*O*-methyltransferase; HUNT: "Helseundersøkelsen i Nord-Trøndelag" (The Nord-Trøndelag Health Study); Val: Valine; Met: Methionine; HADS: Hospital Anxiety and Depression Scale; HADS-A: Anxiety subscale of Hospital Anxiety and Depression Scale; HADS-D: Depression subscale of Hospital Anxiety and Depression Scale.

## Competing interests

The authors declare that they have no competing interests.

## Authors' contributions

KH and PMB conceived of the study and performed the statistical analysis. FS, ES and J–AZ all participated in the design and drafted the manuscript. All authors read and approved the final manuscript.

## Pre-publication history

The pre-publication history for this paper can be accessed here:



## References

[B1] Lotta T, Vidgren J, Tilgmann C, Ulmanen I, Melén K, Julkunen I, Taskinen J (1995). Kinetics of human soluble and membrane-bound catechol-O-methyltransferase: a revised mechanism and description of the thermolabile variant of the enzyme. Biochemistry.

[B2] Craddock N, Owen MJ, O'Donovan MC (2006). The catechol-O-methyl transferase (COMT) gene as a candidate for psychiatric phenotypes: evidence and lessons. Mol Psychiatry.

[B3] Massat I, Souery D, Del-Favero J, Nothen M, Blackwood D, Muir W, Kaneva R, Serretti A, Lorenzi C, Rietschel M, Milanova V, Papadimitriou GN, Dikeos D, Van Broekhoven C, Mendlewicz (2005). Association between COMT (Val158Met) functional polymorphism and early onset in patients with major depressive disorder in a European multicenter genetic association study. Mol Psychiatry.

[B4] Ohara K, Nagai M, Suzuki Y, Ohara K (1998). Low activity allele of the catechol-o-methyltransferase gene and Japanese unipolar depression. Neuroreport.

[B5] Rothe C, Koszycki D, Bradwejn J, King N, Deluca V, Tharmalingam S, Macciardi F, Deckert J, Kennedy JL (2006). Association of the Val158Met catechol-O-methyltransferase genetic polymorphism with panic disorder. Neuropsychopharmacology.

[B6] Samochowiec J, Hajduk A, Samochowiec A, Horodnicki J, Stepien G, Grzywacz A, Kucharska-Mazur J (2004). Association of MAO-A, COMT, and 5-HTT genes polymorphisms in patients with anxiety disorders of the phobic spectrum. Psychiatry Res.

[B7] Woo JN, Yoon KS, Choi YH, Oh KS, Lee YS, Yu BH (2004). The association between panic disorder and the L/L genotype of catechol-O-methyltransferase. J Psychiatr Res.

[B8] Domschke K, Deckert J, O'Donovan MC, Glatt SJ (2007). Meta-Analysis of COMT val158met in Panic Disorder: Ethnic Heterogeneity and Gender Specificity. Am J Med Genet B Neuropsychiatr Genet.

[B9] Spinhoven PH, Ormel J, Sloekers PP, Kempen GI, Speckens AE, Van Hemert AM (1997). A validation study of the Hospital anxiety and depression scale (HADS) in different groups of Dutch subjects. Psychol Med.

[B10] Hermann C (1997). International experiences with the Hospital and depression scale: A review of validation data and clinical results. J Psychosom Res.

[B11] Bjelland I, Dahl AA, Haug TT, Neckelman D (2002). The validity of the Hospital Anxiety and Depression Scale. An updated literature review. J Psychosom Res.

[B12] Aylard PR, Gooding JH, McKenna PJ, Snaith RP (1987). A validation study of three anxiety and depression self-assessment scales. J Psychosom Res.

[B13] Lewis G, Wessely S (1990). Comparison of the General Health Questionnaire and the Hospital Anxiety and Depression Scale. Br J Psychiatry.

[B14] Watson D, Weber K, Assenheimer JS, Clark LA, Strauss ME, McCormick RA (1995). Testing a tripartite model: I. Evaluating the convergent and discriminant validity of anxiety and depression symptom scales. J Abnorm Psychol.

[B15] Lisspers J, Nygren A, Söderman E (1997). Hospital Anxiety and Depression Scale (HAD): some psychometric data for a Swedish sample. Acta Psychiatr Scand.

[B16] Holmen J, Midthjell K, Krüger Ø, Langhammer A, Holmen TL, Bratberg GH, Vatten L, Lund-Larsen PG (2003). The Nord-Trøndelag Health Study 1995–97 (HUNT 2): Objectives, contents, methods and participation. Norsk Epidemiologi.

[B17] Holmen J, Midthjell K, Forsèn L, Skjerve K, Gorseth M, Oseland A (1990). A health survey in Nord-Trøndelag 1984–86. Participation and comparison of attendants and non-attendants. Tidsskr Nor Laegeforen.

[B18] Hagen K, Pettersen E, Stovner LJ, Skorpen F, Holmen J, Zwart JA (2007). High systolic blood pressure is associated with Val/Val genotype in the catechol-o-methyltransferase gene. Am J Hypertens.

[B19] Wittwer CT, Ririe KM, Andrew RV, David DA, Gundry RA, Balis UJ (1997). The LightCycler: a microvolume multisample fluorimeter with rapid temperature control. Biotechniques.

[B20] Stordal E, Krüger MB, Dahl NH, Krüger Ø, Mykletun A, Dahl AA (2001). Depression in relation to age and gender in the general population: the Nord-Trøndelag Health Study (HUNT). Acta Psychiatr Scand.

[B21] Zigmond AS, Snaith RP (1983). The hospital anxiety and depression scale. Acta Psychiatr Scand.

[B22] Stordal E, Mykletun A, Dahl AA (2003). The association between age and depression in the general population: a multivariate examination. Acta Psychiatr Scand.

[B23] Palmatier MA, Kang AM, Kidd KK (1999). Global variation in the frequencies of functionally different catechol-O-methyltransferase alleles. Biol Psychiatry.

[B24] Ohara K, Nagai M, Suzuki Y, Ochiai M, Ohara K (1998). No association between anxiety disorders and catechol-O-methyltransferase polymorphism. Psychiatry Res.

[B25] McGrath M, Kawachi I, Ascherio A, Colditz GA, Hunter DJ, De Vivo I (2004). Association between catechol-O-methyltransferase and phobic anxiety. Am J Psychiatry.

[B26] Domschke K, Freitag CM, Kuhlenbaumer G, Schirmacher A, Sand P, Nyhuis P, Jacob C, Fritze J, Franke P, Rietschel M, Garritsen HS, Fimmers R, Nothen MM, Lesch KP, Stogbauer F, Deckert J (2004). Association of the functional V158M catechol-O-methyltransferase polymorphism with panic disorder in women. Int J Neuropsychopharmacol.

[B27] Freitag CM, Domschke K, Rothe C, Lee YJ, Hohoff C, Gutknecht L, Sand P, Fimmers R, Lesch KP, Deckert J (2006). Interaction of serotonergic and noradrenergic gene variants in panic disorder. Psychiatr Genet.

[B28] Hansen V, Jacobsen K, Arnesen E (2001). Prevalence of Serious Psychiatric Morbidity in Attenders and Nonattenders to a Health Survey of a General Population. Am J Epidemiol.

[B29] Mykletun A, Stordal E, Dahl AA (2001). The Hospital Anxiety and Depression (HAD) scale: factor structure, item analyses, and internal consistency in a large population. Br J Psychiatry.

[B30] Zwart JA, Dyb G, Hagen K, Dahl AA, Ødegård KJ, Bovim G, Stovner LJ (2003). Depression and anxiety disorders associated with headache frequency. The Nord-Trøndelag Health Study. Eur J Neurol.

[B31] Oedegaard KJ, Neckelmann D, Mykletun A, Dahl AA, Zwart JA, Hagen K, Fasmer OB (2006). Migraine with and without aura: Association with depression and anxiety disorder in a population-based study. The HUNT Study. Cephalalgia.

[B32] Kunugi H, Vallada HP, Hoda F, Kirov G, Gill M, Aitchison KJ, Arranz MJ, Murray RM, Collier DA (1997). No evidence for an association of affective disorders with high- or low-activity allele of catechol-o-methyltransferase gene. Biol Psychiatry.

[B33] Frisch A, Postilnick D, Rockah R, Michaelovsky E, Postilnick S, Birman E, Laor N, Rauchverger B, Kreinin A, Poyurovsky M, Schneidman B, Modai I, Weizman R (1999). Association of unipolar major depressive disorder with genes of the seretonergic and dopaminergic pathways. Mol Psychiatry.

[B34] Jabbi M, Kema IP, Pompe G van der, te Meerman GJ, Ormel J, den Boer JA (2007). Catechol-o-methyltransferase polymorphism and susceptibility to major depressive disorder modulates psychological stress response. Psychiatr Genet.

[B35] Wichers M, Aguilera M, Kenis G, Krabbendam L, Myin-Germeys I, Jacobs N, Peeters F, Derom C, Vlietinck R, Mengelers R, Delespaul P, van Os J (2007). The Catechol-O-Methyltransferase Val158Met Polymorphism and Experience of Reward in the Flow of Daily Life. Neuropsychopharmacology.

[B36] Enoch MA, Xu K, Ferro E, Harris CR, Goldman D (2003). Genetic origins of anxiety in women: a role for a functional catechol-O-methyltransferase polymorphism. Psychiatr Genet.

[B37] Martin CR (2005). What does the Hospital anxiety and depression scale (HADS) really measure in liaison psychiatry settings?. Curr Psychiatry Rev.

[B38] Hamilton SP, Slager SL, Heiman GA, Deng Z, Haghighi F, Klein DF, Hodge SE, Weissman MM, Fyer AJ, Knowles JA (2002). Evidence for a susceptibility locus for panic disorder near the catechol-O-methyltransferase gene on chromosome 22. Biol Psychiatry.

[B39] Funke B, Malhotra AK, Finn CT, Plocik AM, Lake SL, Lencz T, DeRosse P, Kane JM, Kucherlapati R (2005). COMT genetic variation confers risk for psychotic and affective disorders: a case control study. Behav Brain Funct.

